# 表皮生长因子受体基因突变非小细胞肺癌的靶向治疗及其耐药机制

**DOI:** 10.3779/j.issn.1009-3419.2022.101.05

**Published:** 2022-03-20

**Authors:** 珺霞 黄, 红 王

**Affiliations:** 1 510515 广州，南方医科大学第二临床医学院 The Second School of Clinical Medicine, Southern Medical University, Guangzhou 510515, China; 2 100071 北京，解放军总医院第五医学中心南区肿瘤内科一区 Department of Lung Oncology, Affiliated Hospital of the PLA Military Academy of Medical Sciences, Beijing 100071, China

**Keywords:** 肺肿瘤, 靶向治疗, 表皮生长因子受体, 酪氨酸激酶抑制剂, 耐药, Lung neoplasms, Targeted therapy, Epidermal growth factor receptor, Tyrosine kinase inhibitors, Drug resistance

## Abstract

肺癌是全球第六大死因，也是恶性肿瘤的主要死因之一，非小细胞肺癌（non-small cell lung cancer, NSCLC）是其最常见的类型。表皮生长因子受体（epidermal growth factor receptor, *EGFR*）基因突变是NSCLC的常见突变之一。针对*EGFR*基因突变的晚期NSCLC患者，使用EGFR-酪氨酸激酶抑制剂（EGFR-tyrosine kinase inhibitors, EGFR-TKIs）如吉非替尼、阿法替尼、奥希替尼等靶向治疗已经成为许多指南推荐的一线治疗方案，但许多患者在用药约1年左右便发生获得性耐药。出现获得性耐药的患者会比普通患者更早出现疾病进展，对患者的预后造成重大影响。目前对于发生了获得性耐药的患者主要的治疗方式为针对耐药突变进行新的靶点抑制，如发生了T790M突变的患者对第一、第二代药物（如吉非替尼、阿法替尼等）产生耐药，可以使用第三代药物（奥希替尼或阿美替尼）进行治疗，通过这种治疗方式可延缓病情进展。因此，耐药机制的研究及耐药患者的治疗是必不可少的。本文主要就EGFR突变NSCLC患者的靶向治疗及其耐药机制进行综述，以期为EGFR-TKIs的临床应用提供参考。

## Abstract (en)

Lung cancer is the sixth leading cause of death worldwide and one of the leading cause of death from malignant tumors. Non-small cell lung cancer (NSCLC) is the most common type of lung cancer. Epidermal growth factor receptor (*EGFR*) gene mutation is a common mutation in NSCLC. For advanced NSCLC patients with *EGFR* mutations, EGFR-tyrosine kinase inhibitors (EGFR-TKIs), such as Gefitinib, Afatinib, Oxitinib and other targeted therapies have become the first-line treatment recommended by many guidelines, but many patients develop acquired drug resistance after about 1 year of medication. Patients with drug resistance will have earlier disease progression than patients without drug resistance, which has an important impact on the prognosis of patients. At present, the main treatment for patients with acquired resistance is new target inhibition for resistant mutation. For example, if patients with T790M mutation are resistant to the first or second generation drugs such as Gefitinb and Afatinib, they can be treated with the third generation drugs (Osimertinib or Almonertinib), which can delay the progression of the disease. Therefore, the study of drug resistance mechanism and treatment of drug resistance patients are essential. This paper mainly reviews targeted therapy and drug resistance mechanism of *EGFR*-mutant NSCLC patients, in order to provide reference for clinical application of EGFR-TKIs.

世界卫生组织关于2020年全球主要死因的调研结果^[1]^中，肺癌位居世界第六，其死亡率在恶性肿瘤中排名第一（18.0%），发病率（11.4%）仅次于乳腺癌（11.7%）。非小细胞肺癌（non-small cell lung cancer, NSCLC）是最常见的肺癌类型，占所有肺癌的80%-90%。

表皮生长因子受体（epidermal growth factor receptor, *EGFR*）基因突变作为NSCLC常见的突变之一（约占NSCLC患者的1/3）^[2]^，常作为靶向治疗的靶点。其靶向药物EGFR-酪氨酸激酶抑制剂（EGFR-tyrosine kinase inhibitors, EGFR-TKIs）被多个指南推荐作为*EGFR*突变晚期NSCLC患者的一线治疗，但是许多患者治疗后10个月-15个月便出现疾病进展，其主要原因是患者发生了获得性耐药。目前对于发生获得性耐药的患者的进一步治疗方式主要是针对耐药突变基因的靶向治疗，此外还有一些关于靶向治疗联合其他治疗方式来减缓耐药的研究。本文主要对*EGFR*突变NSCLC的靶向治疗及其耐药机制研究进行综述，以期为临床治疗提供适当的参考。

## 1 *EGFR*及其基因突变

关于EGFR的报道最早是1980年^[3]^，发现佛波酯（12-0-tetradecanoylphorbol-13-acetate, TPA）可以通过与特定的高亲和力细胞表面膜受体结合起作用，这会导致膜磷脂组成的改变，从而致癌。1985年Hunts^[4]^发现了*EGFR*基因在人类鳞状细胞癌的扩增，1986年Yokota等^[5]^在腺癌中发现其表达扩增，1993年在NSCLC中发现其突变^[6]^。此外，在人表皮细胞癌^[7]^、胶质母细胞瘤^[8]^、乳腺癌^[9]^、食管癌^[10]^、胃癌^[11]^、结肠癌^[12]^等肿瘤中也相继发现了EGFR的表达异常。

### 1.1 EGFR及其功能

表皮生长因子（epidermal growth factor, EGF）是最早发现的生长因子之一，属于多肽家族^[13]^，与其受体EGFR结合可诱导哺乳动物细胞的增殖或分化^[14]^。

EGFR是由1, 186个氨基酸组成的约170 kDa的单链氨基酸多肽链^[15]^，属于表皮生长因子受体家族（包含EGFR/ERBB-1/HER1、HER2/ERBB-2/Neu、HER3/ERBB-3、HER4/ERBB-4四类，分别由ERBB1-4编码）^[16, 17]^，表达于大多数正常细胞表面。该受体主要由胞外域（细胞外配体结合区域）、胞内域（具有酪氨酸酶活性）及跨膜区（单一疏水锚定序列）构成^[18]^。EGFR与配体结合后被激活，通过Ras/Raf/MEK/ERK/MAPK通路、PI3K/AKT（PKB）通路^[17]^及JAK/STAT通路^[19]^等信号通路进行信息传递，从而影响肿瘤细胞增殖、血管生成、侵袭和转移。

### 1.2 *EGFR*编码基因及其突变

*EGFR*的编码基因*ERBB*来源于与其相应受体相关的禽流感病毒——成红细胞病致癌基因的名称。EGFR包括ERBB、ERBB1和HER1。ERBB1指成红细胞病病毒，HER1指人EGFR受体1^[17]^。*EGFR*基因位于第7号染色体短臂^[20]^，与之有关的突变在NSCLC中共发现200多种，主要发生在外显子18-外显子21，最常见的突变为外显子19的框内缺失（19del）及外显子21的单点突变（L858R），占*EGFR*突变的80%以上^[21, 22]^。

## 2 EGFR-TKIs

EGFR-TKIs主要通过与EGFR结合发挥抑制肿瘤增殖及侵袭的作用。

### 2.1 第一代EGFR-TKIs

第一代EGFR-TKIs主要通过抑制EGFR酪氨酸的自体磷酸化，从而抑制下游信号传导，阻止癌细胞增殖。其代表药物包括吉非替尼（Gefitinib）、厄洛替尼（Erotinib）、埃克替尼（Icotinib）等。

#### 2.1.1 吉非替尼（易瑞沙，伊瑞可，Gefitinib）

吉非替尼是由英国公司阿斯利康（AstraZeneca）公司研制开发的一种特异性较高的抗肿瘤靶向药物，是首个EGFR-TKI，2003年经美国食品药品监督管理局（Food and Drug Administration, FDA）批准用于铂类药物和多西他赛化疗后疾病进展的NSCLC，是第一个用于治疗NSCLC的分子靶向药物。

与标准化疗相比，吉非替尼用于敏感性*EGFR*突变（外显子19del、外显子21L858R简称L858R）晚期NSCLC患者的一线治疗可以延长患者的无进展生存期（progression-free survival, PFS）（吉非替尼*vs*化疗，中位PFS：10.8个月*vs* 5.4个月）和总生存期（overall survival, OS）（OS：30.5个月*vs* 23.6个月），其安全性好，毒性可耐受——其最常见的不良事件（adverse event, AE）为皮疹（71.1%）、转氨酶升高（55.3%）及腹泻（46.6%）^[23, 24]^。

*EGFR*突变晚期NSCLC患者一线使用吉非替尼联合铂类化疗较单用吉非替尼的客观缓解率（objective response rate, ORR）更高（84% *vs* 67%, *P* < 0.001），且PFS更长[中位PFS（median PFS, mPFS）：20.9个月*vs* 11.2个月]，可能会延缓晚期*EGFR*突变（外显子19del、L858R、G719A、G719C、L861Q）NSCLC患者对于EGFR-TKIs的耐药，且患者对其反应率更高，但联合治疗的AE大于单药治疗（3级以上AE：65.3% *vs* 31.2%），不过大多数毒性反应如中性粒细胞减少症、贫血及血小板减少等是可控的^[25]^。

还有研究^[26]^表明，吉非替尼与特泊替尼（Tepotinib）联用可以提高其在*EGFR*突变NSCLC和*MET*扩增患者中的抗肿瘤活性。*EGFR*突变NSCLC合并有其他突变时，可以采用两种或多种靶向药物联合治疗，不过AE发生的概率可能会更高，可根据患者的病情决定治疗方案。

#### 2.1.2 厄洛替尼（特罗凯，Erlotinib，OSI-774）

厄洛替尼是由Genentech公司和OSI公司联合研发，2004年被FDA批准上市，2005年被FDA批准与吉西他滨作为胰腺癌一线治疗。2010年，经FDA批准用于局部晚期或转移性NSCLC的一线维持治疗。

厄洛替尼一线治疗*EGFR*外显子19del、21L858R突变的NSCLC患者，可以延长患者的mPFS^[27]^，其ORR及mPFS均优于化疗（ORR：62.7% *vs* 33.6%；mPFS：11.0个月*vs* 5.5个月），且其发生严重AE的概率更小（2.7% *vs* 10.6%），最常见的3级以上AE为皮疹（6.4%）^[28]^。

厄洛替尼联合贝伐珠单抗治疗*EGFR*突变（外显子19del、外显子21L858R）晚期NSCLC患者的mPFS为16.9个月，95%可信区间（confidence interval, CI）为14.2个月-21.0个月，优于单用厄洛替尼（mPFS为13.3个月），但两者联合治疗引起的3级以上AE比单药治疗更高（88% *vs* 46%），以皮疹为主（21%），最常见的严重AE为4级中性粒细胞减少症（2%）和4级肝功能障碍（1%）^[29]^。使用厄洛替尼作为辅助治疗可以改善*EGFR*突变NSCLC患者的2年无病生存期（disease-free survival, DFS），并提高患者的根治性切除率^[30]^。此外，厄洛替尼与纳武单抗联用可能使厄洛替尼耐药的晚期*EGFR*突变NSCLC患者获益，并且其毒性可耐受^[31]^。目前暂不清楚厄洛替尼与哪些药物联合使用可减缓耐药的形成。

#### 2.1.3 埃克替尼（凯美纳，Icotinib）

埃克替尼是由我国浙江贝达药业有限公司研发，2011年6月7日获得国家食品药品监督管理总局（China Food and Drug Administration, CFDA）批准上市，并用于*EGFR*突变的局部晚期或转移性NSCLC患者的一线治疗。埃克替尼能够抑制A549细胞（一种NSCLC细胞系）增殖，加速其死亡；并可通过调节上皮细胞间质转化（epithelial-mesenchymal transition, EMT）相关蛋白的表达介导A549细胞中EMT的进程，从而降低A549细胞迁移和侵袭的能力^[32]^。

晚期肺腺癌患者一线使用埃克替尼的PFS相较于化疗组更长（11.2个月*vs* 7.9个月），其常见的3级或4级AE是皮疹（14.8%）和腹泻（7.4%），其安全性较化疗更好，且可耐受^[33]^。研究^[34, 35]^表明，大剂量埃克替尼（250 mg *tid*）可改善L858R突变NSCLC患者的mPFS（250 mg *vs* 125 mg：12.9个月*vs* 9.2个月）和ORR（75% *vs* 48%）。此外，埃克替尼显著提高了完全切除肿瘤后*EGFR*突变II期-IIIa期NSCLC患者的DFS（埃克替尼*vs*化疗：47.0个月*vs* 22.1个月）。

埃克替尼联合化疗作为一线治疗可显著提高敏感性*EGFR*突变（外显子19del、外显子21L858R）晚期肺腺癌患者的PFS（联合*vs*单用埃克替尼：16.0个月*vs* 10.0个月），其ORR和疾病控制率（disease control rate, DCR）也高于单用埃克替尼治疗组（ORR: 77.8% *vs* 64.0%; DCR: 91.1% *vs* 79.8%），但两组的OS没有明显差异（36.0个月*vs* 34.0个月），且联合治疗所引起的3级及以上AE（白细胞减少、肝功能损害）更高^[36]^，对于敏感性*EGFR*突变的肺腺癌患者，可根据患者病情决定是否联合治疗。

### 2.2 第二代EGFR-TKIs

#### 2.2.1 阿法替尼（吉泰瑞，Afatinib，BIBW 2992）

阿法替尼是由德国勃林格殷格翰公司（Boehringer Ingelheim）开发的第二代EGFR及HER2酪氨酸激酶的双重抑制剂，也是第一个获批的不可逆ERBB系列阻断剂，能不可逆地阻断EGFR及HER2酪氨酸激酶的过表达，从而阻断癌细胞信号传导。2013年经FDA批准用于治疗NSCLC。

在LUX-Lung 7的研究中发现：与第一代药物吉非替尼相比，阿法替尼可显著改善*EGFR*突变（外显子19del、外显子21L858R）NSCLC患者的PFS（11.0个月）、治疗失败时间（time to failure, TTF）及ORR（70%），但不能改善患者的中位OS^[37]^。除了对外显子19del具有良好的活性，对于一些罕见突变（如G719X、G719A、L861Q、S768I）、复合*EGFR*突变及外显子20ins，阿法替尼同样具有广泛的活性^[38-40]^，并且能显著提高脑转移患者的ORR^[41]^。其常见的3级及以上AE为腹泻（19.4%）、甲沟炎（16%）及皮疹（46.1%）^[38, 42]^。

阿法替尼联合铂类双药（卡铂、培美曲塞）化疗治疗第一代EGFR-TKIs治疗后进展的NSCLC患者的总反应率为30%，mPFS达13.7个月，剂量限制性毒性包括3级腹泻、3级低钾血症、3级血清淀粉酶升高和4级血小板减少症，总体来说耐受性及临床疗效较好^[43]^。此外，有研究^[44]^表明阿法替尼联合西妥昔单抗对于鳞状NSCLC患者具有一定的抗肿瘤活性（75%为SD），常见AE为腹泻及痤疮样皮炎。阿法替尼联合贝伐珠单抗可改善*EGFR*突变（外显子19del、外显子21L858R）的晚期NSCLC患者的预后，mPFS为24.2个月，3级AE为腹泻及皮疹^[45]^。

#### 2.2.2 达克替尼（多泽润，Dacomitinib，Vizimpro）

达克替尼是美国辉瑞公司（Pfizer）研制的第二代、不可逆的EGFR-TKIs，是一种多激酶受体抑制剂，能不可逆抑制3种不同ERBB家族分子成员，包括EGFR（HER1）、HER2、HER4。2018年，经FDA批准作为一线疗法治疗*EGFR*基因外显子19del或外显子21L858R点突变的转移性NSCLC患者^[46]^。

达克替尼较吉非替尼相比，可以显著改善EGFR+ 晚期NSCLC患者的mPFS（14.7个月*vs* 9.2个月）及OS (34.1个月*vs* 26.8个月)，其主要AE为腹泻（87%）、甲沟炎（62%）、痤疮性皮肤炎（49%）、口腔炎（44%），最常见的3级以上AE为痤疮性皮炎（27.5%）^[47, 48]^。

### 2.3 第三代EGFR-TKIs

#### 2.3.1 奥希替尼（泰瑞沙，Osimertinib，AZD-9291）

奥希替尼是由英国阿斯利康（AstraZeneca）公司研制开发第三代治疗NSCLC的EGFR-TKIs。2015年奥希替尼经FDA批准用于NSCLC，2018年批准其用于一线治疗*EGFR*外显子19del或外显子21L858R突变的转移性NSCLC患者，2020年批准其用于治疗手术切除后的*EGFR*外显子19del或外显子21L858R突变的NSCLC患者。

奥西替尼可以延长*EGFR*突变（外显子19del、外显子21L858R）NSCLC患者的DFS^[49]^。对于一线EGFR-TKIs治疗期间疾病进展的T790M突变晚期NSCLC患者，奥希替尼的疗效明显优于传统化疗（PFS：10.1个月*vs* 4.4个月；ORR：71% *vs* 31%）^[50]^。DCR为91.1%，mPFS为11.0个月。其最常见的AE为腹泻（41%）、贫血（37.5%）、皮肤毒性（如皮疹、甲沟炎等，占35.7%）^[50, 51]^。

此外，对于合并有*MET*扩增的*EGFR*突变NSCLC患者，联合使用奥希替尼和萨沃利替尼（又称沃利替尼，一种MET-TKI）可以使其得到一定获益^[52]^。

#### 2.3.2 艾维替尼（Avitinib, AC0010）

艾维替尼是浙江艾森医药公司自主研发的国内首个第三代EGFR-TKIs，靶向*EGFR*敏感突变（外显子19del、外显子21L858R）和T790M突变，用于治疗*EGFR*突变或耐药突变的NSCLC。艾维替尼可以保留野生型*EGFR*，通过与ATP结合口袋中的CYS797形成共价键从而不可逆地结合EGFR，能克服T790M诱导的耐药性^[53]^。

*EGFR*-T790M突变NSCLC患者对于艾维替尼有良好的耐受性，其mPFS为247 d（8.2个月），OS为536 d（17.9个月），脑转移患者的mPFS为142 d（4.7个月），其中最常见的AE是轻度的可逆性转氨酶升高（62.5%）和腹泻（25%）。艾维替尼对血脑屏障（blood brain barrier, BBB）渗透性低（BBB渗透率为0.046%-0.146%），但对无症状脑转移瘤有较好的控制作用^[54]^。其常见的AE为腹泻（75%）、皮疹（48%）和丙氨酸转氨酶水平升高（44%）^[55]^。

#### 2.3.3 阿美替尼（阿美乐，Almonertinib，HS-10296）

阿美替尼（甲磺酸阿美替尼）是由江苏豪森药业公司研发的第三代EGFR-TKIs，靶向敏感性*EGFR*突变（外显子19del、L858R）和T790M突变，用于治疗既往使用EGFR-TKIs治疗时或治疗后出现疾病进展且*EGFR*-T790M突变阳性的局部晚期或转移性NSCLC成人患者。

在一项多中心临床研究^[56]^中，使用阿美替尼的T790M突变NSCLC患者ORR为52%，DCR为92%，mPFS为11.0个月。3级或以上的AE主要是血肌酸磷酸激酶升高（10%）和丙氨酸氨基转移酶升高（3%），其安全性和耐受性良好。目前，关于阿美替尼的临床研究较少，对于其耐药情况、治疗相关AE、其他联合治疗措施对患者病情改善的程度均不明，还需要大量临床研究来明确。

#### 2.3.4 艾氟替尼（Aflutinib，伏美替尼，AST2818）

艾氟替尼是一种基于三氟乙氧基吡啶的不可逆EGFR-TKIs，是由上海艾力斯医药科技有限公司研制的第三代EGFR-TKIs，用于治疗敏感性*EGFR*突变（外显子19del、L858R、L861Q）及耐药突变（G719X、T790M）的NSCLC患者。

艾氟替尼在治疗T790M突变NSCLC患者中，每日剂量高达240 mg时仍可耐受，但治疗剂量大于80 mg可能不会对NSCLC有更加显著的抗肿瘤活性。其代谢产物AST5902仍具有药理活性，并且其血脑渗透率好，对于颅内病变治疗有效。其中位治疗持续时间为7.4个月，主要的AE为皮疹（10%）、痤疮样皮炎（6%）、腹泻（19%）、恶心（7%）、呕吐、口腔炎及便秘（各占5%）。总体耐受性较好^[57]^。

在一项多中心、单臂的IIb期临床研究^[58]^中，共纳入了220例T790M突变NSCLC患者，每日口服80 mg艾氟替尼中位随访时间为9.6个月，ORR为74%（163/220）。治疗期间，共有58例患者（26%）发生了3级及以上AE，主要为γ-谷氨酰转移酶升高（2%）、转氨酶升高（1%）、低钠血症（1%）、高血压（1%）、肺部感染（1%）、高镁血症（1%）和心包积液（1%）。其他级别的AE主要为腹泻（5%）及皮疹（7%）。总体来说，艾氟替尼的疗效及安全性好。

### 2.4 第四代*EGFR*-TKIs

#### 2.4.1 EAI045

EAI045是一种变构抑制剂，抑制*EGFR* L858R/T790M突变。作为单一药物，它不能有效阻断细胞中EGFR驱动的细胞增殖，即没有抗肿瘤作用。EAI045与西妥昔单抗联合在由*EGFR*（L858R/T790M）和*EGFR*（L858R/T790M/C797S）驱动的肺癌小鼠模型中有效，*EGFR*（L858R/T790M/C797S）是一种对所有当前可用的EGFR-TKIs具有抗性的突变体^[59]^，但其不能抑制*EGFR*-del19/T790M/C797S突变。

#### 2.4.2 CH7233163

CH7233163是一种非共价ATP竞争性抑制剂，选择性抑制*EGFR*-del19/T790M/C797S，在体内外对*EGFR*-del19/T790M/C797S均显示出有效的抗肿瘤活性。此外，它还能选择性地抑制各种类型的*EGFR*突变体（例如，L858R/T790M/C797S、L858R/T790M、del19/T790M、L8野生型）。由于CH7233163可以抑制多种*EGFR*突变，可能降低对靶向治疗的耐药，使用CH7233163治疗对奥希替尼耐药的患者（尤其是有*EGFR*-del19/T790M/C797S突变）可能有益^[60]^。

#### 2.4.3 TQB3804

TQB3804是我国正大天晴药业公司研发的第四代EGFR-TKIs，可克服C797S突变和T790M突变。目前暂无发表相关临床研究文献。在临床试验方面，有两项关于TQB3804正在进行的临床研究：NCT04128085（I期）、NCT04180150（II期）。

目前暂时还没有针对EAI045、CH7233163的临床研究。第四代EFGR-TKIs缺乏大量临床研究证实其有效性及不良反应，需等待临床实践进一步证实。

## 3 EGFR-TKIs耐药与耐药后治疗

总的来说，EGFR-TKIs对于NSCLC的治疗效果明显优于化疗，但无法使NSCLC患者获得根治，在带来中位9.2个月-19.3个月的PFS后，会因为耐药的出现而引起疾病进展^[24, 27, 28, 30]^。所以，近年来有大量研究^[24, 27, 30, 38, 40, 61-63]^对EGFR-TKIs的耐药机制进行了报道。总之，引发EGFR-TKIs获得性耐药的机制主要分为EGFR依赖型和EGFR非依赖型，其中EGFR非依赖型耐药机制包括替代途径激活和组织学或表型转化。

### 3.1 EGFR依赖型耐药机制及耐药后治疗

#### 3.1.1 T790M突变

第一、第二代EGFR-TKIs获得性耐药最常见的原因为*EGFR*-T790M突变，占50%-60%^[64, 65]^。T790M突变是指在外显子20上第790位氨基酸由苏氨酸变成甲硫氨酸。当发生*EGFR*-T790M突变时，位于EGFR蛋白ATP结合口袋内的T790M残基会增强蛋白质对ATP的亲和力，以此介导TKI抗性，从而降低其疗效^[66]^。第一代、第二代EGFR-TKIs联合化疗可能减缓发生T790M突变，减慢耐药的发生。

针对*EGFR*-T790M突变，目前最主要的治疗方式是使用第三代药物奥希替尼、艾氟替尼及阿美替尼等治疗防止疾病进展或出现无法耐受的AE。当第三代EGFR-TKIs奥希替尼二线治疗出现疾病进展时，约有一半的患者会出现*EGFR*-T790M缺失，并且通常会伴随EGFR非依赖型耐药机制的产生，比如*MET*/*HER2*扩增、*KRAS*突变、小细胞转化和基因融合等^[67, 68]^。此时，需要适当联合其他靶向药物（如联合MET抑制剂）治疗合并*MET*扩增的NSCLC患者。

#### 3.1.2 *EGFR*-C797S突变、*EGFR*-L792F突变

*EGFR*-C797S被认为是第三代EGFR-TKIs的主要耐药机制，是奥希替尼结合位点发生突变，导致第797位氨基酸由半胱氨酸转变成丝氨酸，导致药物共价结合受阻，从而引起耐药^[69]^。*EGFR*-C797S突变占二线奥希替尼耐药病例的10%-25%，约占一线奥希替尼耐药病例的7%^[70]^。但是，*EGFR*-C797S突变与T790M呈反式结构时，会表现出对第一代联合第三代EGFR-TKIs敏感；相反，若二者呈顺式结构，则仍然表现出耐药^[71-73]^。

针对*EGFR*-C797S突变、*EGFR*-L792F突变，主要使用第四代药物EAI045、CH7233163等治疗。

#### 3.1.3 PI3K/Akt/mTOR通路激活

PI3K是细胞内脂质激酶家族的成员，可磷酸化磷脂酰肌醇和磷酸肌醇的3-羟基。*PIK3CA*是一种编码p110α催化亚基的基因，催化亚基p110α与调节亚基p85共同构成IA型磷脂酰肌醇3激酶α（PI3Kα）。PIK3α可以将磷脂酰肌醇（PIP_2_）磷酸化为三磷酸-磷脂酰肌醇（PIP_3_），后者可以激活PI3K/Akt/mTOR信号通路，从而调整肿瘤细胞增殖能力^[74]^。*PI3KCA*基因突变或扩增可引发PI3K/Akt/mTOR通路异常激活。*PI3KCA*突变通常与其他驱动突变（*EGFR*和*KRAS*突变）共同发生，患者预后通常较差^[75]^。*PIK3CA*一些突变与二线奥希替尼耐药相关，发生率为4%-11%，其中包括E545K、E542K、R88Q、N345K和E418K突变^[70]^。

针对PI3K/Akt、mTOR通路激活，可予以第四代药物BLU-945治疗。此外，PI3K抑制剂（LY294002）与EGFR-TKIs联合使用有可能提高EGFR-TKI的敏感性^[76]^，联合治疗对于患者效果可能更好，需要临床试验进一步证明。

#### 3.1.4 其他罕见突变

在临床研究中，发现D761Y、T854A突变^[77]^、L747S^[78]^也与EGFR-TKIs的耐药有关。L747S突变发生在h3链与α-C-螺旋之间的环的起始处，D761Y突变位于α-C-螺旋中，T854A位于EGFR的激活环中。其残基位置（L747、D761和T854）靠近ATP或可逆EGFR-TKI的结合位点，突变后影响ATP或可逆EGFR-TKI的亲和力^[79]^，降低TKI疗效。此外，在阿法替尼的细胞中发现了*EGFR*-L792F突变，具体机制尚不清楚^[40]^。针对这些罕见突变，目前暂时没有明确的治疗方式，还在进一步研究中。

### 3.2 替代途径激活

由*EGFR*下游基因突变、基因融合、基因扩增、编码细胞周期相关基因突变等引起的替代突进激活，属于EGFR非依赖型耐药机制，也与EGFR-TKIs的耐药有关。

#### 3.2.1 *MET*扩增

*MET*扩增为最常见的替代途径激活（旁路途径），占EGFR-TKIs获得性耐药的5%-10%^[80, 81]^，且更常见于传统型*EGFR*突变^[82]^，主要通过驱动ERBB3（HER3）二聚化来激活PI3K-AKT信号通路^[80]^，从而影响肿瘤细胞增殖和转移。*MET*扩增可以引起多种EGFR-TKIs耐药^[80, 83]^。

对于发生*MET*扩增的晚期NSCLC患者，予以MET受体抑制剂卡马替尼治疗可得到一定获益。MET抑制剂Tepotinib联合铂类一起治疗*EGFR*突变合并*MET*扩增的NSCLC患者，其疗效高于化疗^[84]^。MET抑制剂对于*MET*扩增引起的EGFR耐药患者的获益需要进一步研究。

#### 3.2.2 *HER2*扩增

*HER2*基因是位于17号染色体长臂上的（17q21）的原癌基因。其在NSCLC中的发生率为1%-4%，与其他致癌驱动因素（如*EGFR*、*KRAS*、*BRAF*等）相互排斥^[85]^。HER2（Neu）受体是一种具有酪氨酸激酶活性的跨膜糖蛋白，属于EGFR家族。*HER2*扩增后会引起HER2下游信号通路的组成型激活，从而引起HER2过表达，HER2的浓度上升后，可以通过与其配体激活的EGFR或HER3发生异二聚化，或发生同二聚化导致酪氨酸残基磷酸化，从而启动下游信号通路，如PI3K/AKT信号通路、Ras/MEK/ERK和JAK/STAT信号通路，从而调节肿瘤细胞的生长^[86]^。有研究^[82]^发现，扩增是NSCLC发生EGFR-TKIs获得性耐药的机制之一，一线奥西替尼耐药约占2%，二线则为5%。AUR3研究^[68]^显示，在出现奥希替尼耐药的病例中发现*HER2*扩增会与*EGFR*-L792X+C797X+*PIK3CA*扩增（1%）、*EGFR*-G796S+*MET*扩增（1%）和*PIK3CA*扩增（1%）发生共同突变。

针对具有*HER2*突变（尤其是HER2外显子20突变）的肺腺癌患者，使用HER2抑制剂Poziotinib疗效好^[87]^。*HER2*外显子20ins的NSCLC患者对于泛HER受体TKI吡咯替尼敏感^[88]^，使用HER受体抑制剂治疗可能对于*HER2*扩增引起的获得性耐药患者有益。

#### 3.2.3 HER3激活

在EGFR-TKIs耐药的细胞中发现HER3水平更高，HER3过表达（HER3激活）可能与EGFR-TKIs的耐药有关。U3-1402可以抑制EGFR-TKIs的耐药性，联合使用可能使EGFR-TKIs耐药的NSCLC患者获益^[89]^。

#### 3.2.4 RAS-MARK通路激活

RAS-MAPK信号通路中的*KRAS*、*BRAF*和*MAPK1*等基因突变也会引起一线或二线EGFR-TKIs耐药。研究^[90]^报道的耐药相关*KRAS*突变包括G12S、G12D、G13D、Q61R和Q61K。*BRAF* V600E突变引发一线或二线奥希替尼治疗进展均有文献^[91]^报道，并且细胞学试验^[92]^表明，对*EGFR* T790M+*BRAF* V600E突变细胞系使用EGFR和BRAF抑制剂可以有效抑制肿瘤生长。

#### 3.2.5 基因融合

癌基因融合在奥希替尼二线治疗耐药病例中占3%-10%，并且可以和*EGFR*-C797S、*BRAF*突变和*MET*扩增等共同存在^[93]^。目前已经报道的和耐药相关的基因融合包括：*ALK*融合、*GFR3*-*TACC3*、*RET*-*ERC1*、*CCDC6*-*RET*、*NTRK1*-*TPM3*、*NCOA4*-*RET*、*GOPC*-*ROS1*、*AGK*-*BRAF*和*ESYT2*-*BRAF*等^[94, 95]^。

### 3.3 组织学和表型转化

#### 3.3.1 小细胞肺癌（small cell lung cancer, SCLC）转化

有5%-10%的患者在使用EGFR-TKIs治疗后发生从转化为NSCLC到转变为SCLC的组织学转变^[96]^，这种转变会显著影响患者的预后并引发耐药，基因组学研究发现其会发生*RB1*和*TP53*基因失活^[97]^。

#### 3.3.2 EMT

EMT是上皮组织转化成间质的一种细胞程序，对于胚胎发育、伤口愈合和细胞恶变至关重要。EMT由EMT诱导转录因子协调，诱导促进间充质细胞状态的基因表达并抑制维持上皮状态的基因表达^[98]^。HCC827GR细胞（NSCLC细胞系-吉非替尼抗性细胞株）中检出EMT，该类细胞miR-625-3p降低，研究^[99]^认为miR-625-3p/AXL轴通过激活TGF-β/Smad通路促进吉非替尼耐药。此外，还有一些EGFR-TKIs耐药的细胞显现了EMT特征，因此EMT被认为是EGFR-TKIs耐药的可能机制^[100]^。

## 4 总结与展望

目前靶向治疗仍是晚期NSCLC患者的重要治疗措施，能有效延长NSCLC患者的mPFS，并改善其预后，但治疗期间发生获得性耐药是导致疾病进展的主要原因。针对耐药机制进行药品研发，从而对耐药后的患者展开进一步治疗的行动刻不容缓。但治疗后耐药一直是一个亟待解决的问题，对于药品进行大量的临床研究，从而不断发现药品可能存在的耐药机制，对于新药的研发有促成作用。此外，对于新型治疗方法的研究也值得我们进一步探索，包括药物的联合使用，如抗EGFR治疗联合放化疗、手术治疗，抗EGFR治疗联合免疫治疗，旧药联合其他药物使用有时也可以获得意想不到的收获，有些药物联用甚至可以延缓耐药的发生^[101]^。新一代EGFR-TKIs的研究也同样重要，研究耐药基因并根据其对药物进行更新，改善治疗效果。对于*EGFR*耐药基因的研究，可能使得基因治疗成为攻克耐药难题的武器，这也是国内外需要研究的重点难点及治疗前景。综上所述，*EGFR*突变NSCLC患者的治疗离不开EGFR-TKIs及其他治疗手段，科技的进步会不断加深对于*EGFR*耐药基因的研究，基因治疗可能成为根除EGFR-TKIs耐药的措施，甚至是根除癌症的治疗手段。

## References

[1] Sung H, Ferlay J, Siegel RL (2021). Global cancer statistics 2020: GLOBOCAN estimates of incidence and mortality worldwide for 36 cancers in 185 countries. CA Cancer J Clin.

[2] Zhang YL, Yuan JQ, Wang KF (2016). The prevalence of *EGFR* mutation in patients with non-small cell lung cancer: a systematic review and *meta*-analysis. Oncotarget.

[3] Weinstein IB (1980). Cell culture systems for studying multifactor interactions in carcinogenesis. Dev Toxicol Environ Sci.

[4] Hunts J, Ueda M, Ozawa S (1985). Hyperproduction and gene amplification of the epidermal growth factor receptor in squamous cell carcinomas. Jpn J Cancer Res.

[5] Yokota J, Yamamoto T, Toyoshima K (1986). Amplification of c-erbB-2 oncogene in human adenocarcinomas *in vivo*. Lancet.

[6] Garcia DPI, Adams GP, Sundareshan P (1993). Expression of mutated epidermal growth factor receptor by non-small cell lung carcinomas. Cancer Res.

[7] Shimizu N, Kondo I, Gamou S (1984). Genetic analysis of hyperproduction of epidermal growth factor receptors in human epidermoid carcinoma A431 cells. Somat Cell Mol Genet.

[8] Libermann TA, Nusbaum HR, Razon N (1985). Amplification and overexpression of the EGF receptor gene in primary human glioblastomas. J Cell Sci Suppl.

[9] Filmus J, Trent JM, Pollak MN (1987). Epidermal growth factor receptor gene-amplified MDA-468 breast cancer cell line and its nonamplified variants. Mol Cell Biol.

[10] Hollstein MC, Smits AM, Galiana C (1988). Amplification of epidermal growth factor receptor gene but no evidence of *ras* mutations in primary human esophageal cancers. Cancer Res.

[11] Yoshida K, Tsuda T, Matsumura T (1989). Amplification of epidermal growth factor receptor (*EGFR*) gene and oncogenes in human gastric carcinomas. Virchows Arch B Cell Pathol Incl Mol Pathol.

[12] Ravikumar TS, Wolf B, Cocchiaro C (1989). *Ras* gene activation and epidermal growth factor receptor expression in human colon cancer. J Surg Res.

[13] Cohen S (1983). The epidermal growth factor (EGF). Cancer.

[14] Yarden Y, Schlessinger J (1987). Epidermal growth factor induces rapid, reversible aggregation of the purified epidermal growth factor receptor. Biochemistry.

[15] Carpenter G, Cohen S (1990). Epidermal growth factor. J Biol Chem.

[16] Wheeler DL, Huang S, Kruser TJ (2008). Mechanisms of acquired resistance to cetuximab: role of HER (ErbB) family members. Oncogene.

[17] Roskoski RJ (2014). The ErbB/HER family of protein-tyrosine kinases and cancer. Pharmacol Res.

[18] Voldborg BR, Damstrup L, Spang-Thomsen M (1997). Epidermal growth factor receptor (EGFR) and *EGFR* mutations, function and possible role in clinical trials. Ann Oncol.

[19] Leonard WJ, O'Shea JJ (1998). Jaks and STATs: biological implications. Annu Rev Immunol.

[20] Korc M, Meltzer P, Trent J (1986). Enhanced expression of epidermal growth factor receptor correlates with alterations of chromosome 7 in human pancreatic cancer. Proc Natl Acad Sci U S A.

[21] Lynch TJ, Bell DW, Sordella R (2004). Activating mutations in the epidermal growth factor receptor underlying responsiveness of non-small-cell lung cancer to gefitinib. N Engl J Med.

[22] Paez JG, Janne PA, Lee JC (2004). *EGFR* mutations in lung cancer: correlation with clinical response to gefitinib therapy. Science.

[23] Maemondo M, Inoue A, Kobayashi K (2010). Gefitinib or chemotherapy for non-small-cell lung cancer with mutated *EGFR*. N Engl J Med.

[24] Mok TS, Wu YL, Thongprasert S (2009). Gefitinib or carboplatin-paclitaxel in pulmonary adenocarcinoma. N Engl J Med.

[25] Hosomi Y, Morita S, Sugawara S (2020). Gefitinib alone versus gefitinib plus chemotherapy for non-small-cell lung cancer with mutated epidermal growth factor receptor: NEJ009 study. J Clin Oncol.

[26] Wu YL, Cheng Y, Zhou J (2020). Tepotinib plus gefitinib in patients with *EGFR*-mutant non-small-cell lung cancer with MET overexpression or *MET* amplification and acquired resistance to previous EGFR inhibitor (INSIGHT study): an open-label, phase 1b/2, multicentre, randomised trial. Lancet Respir Med.

[27] Kelly K, Altorki NK, Eberhardt WE (2015). Adjuvant erlotinib versus placebo in patients with stage Ib-IIIa non-small-cell lung cancer (RADIANT): A randomized, double-blind, phase III trial. J Clin Oncol.

[28] Wu YL, Zhou C, Liam CK (2015). First-line erlotinib versus gemcitabine/cisplatin in patients with advanced *EGFR* mutation-positive non-small-cell lung cancer: analyses from the phase III, randomized, open-label, ENSURE study. Ann Oncol.

[29] Saito H, Fukuhara T, Furuya N (2019). Erlotinib plus bevacizumab versus erlotinib alone in patients with EGFR-positive advanced non-squamous non-small-cell lung cancer (NEJ026): interim analysis of an open-label, randomised, multicentre, phase 3 trial. Lancet Oncol.

[30] Pennell NA, Neal JW, Chaft JE (2019). SELECT: A phase II trial of adjuvant erlotinib in patients with resected epidermal growth factor receptor-mutant non-small-cell lung cancer. J Clin Oncol.

[31] Gettinger S, Hellmann MD, Chow L (2018). Nivolumab plus erlotinib in patients with *EGFR*-mutant advanced NSCLC. J Thorac Oncol.

[32] Wang GH, Hu ZY (2019). Icotinib inhibits proliferation and epithelial-mesenchymal transition of non-small cell lung cancer A549 cells. Math Biosci Eng.

[33] Shi YK, Wang L, Han BH (2017). First-line icotinib versus cisplatin/pemetrexed plus pemetrexed maintenance therapy for patients with advanced *EGFR* mutation-positive lung adenocarcinoma (CONVINCE): a phase 3, open-label, randomized study. Ann Oncol.

[34] Li X, Zhang L, Jiang D (2020). Routine-dose and high-dose icotinib in patients with advanced non-small cell lung cancer harboring *EGFR* exon 21-L858R mutation: the randomized, phase II, INCREASE trial. Clin Cancer Res.

[35] He J, Su C, Liang W (2021). Icotinib versus chemotherapy as adjuvant treatment for stage II-IIIa *EGFR*-mutant non-small-cell lung cancer (EVIDENCE): a randomised, open-label, phase 3 trial. Lancet Respir Med.

[36] Xu L, Qi Q, Zhang Y (2019). Combination of icotinib and chemotherapy as first-line treatment for advanced lung adenocarcinoma in patients with sensitive *EGFR* mutations: A randomized controlled study. Lung Cancer.

[37] Paz-Ares L, Tan EH, O'Byrne K (2017). Afatinib versus gefitinib in patients with *EGFR* mutation-positive advanced non-small-cell lung cancer: overall survival data from the phase IIb LUX-Lung 7 trial. Ann Oncol.

[38] Kim Y, Lee SH, Ahn JS (2019). Efficacy and safety of afatinib for *EGFR*-mutant non-small cell lung cancer, compared with gefitinib or erlotinib. Cancer Res Treat.

[39] Yang JC, Schuler M, Popat S (2020). Afatinib for the treatment of NSCLC harboring uncommon *EGFR* mutations: A database of 693 cases. J Thorac Oncol.

[40] Kobayashi Y, Azuma K, Nagai H (2017). Characterization of *EGFR* T790M, L792F, and C797S mutations as mechanisms of acquired resistance to afatinib in lung cancer. Mol Cancer Ther.

[41] Schuler M, Wu YL, Hirsh V (2016). First-line afatinib versus chemotherapy in patients with non-small cell lung cancer and common epidermal growth factor receptor gene mutations and brain metastases. J Thorac Oncol.

[42] Wu YL, Sequist LV, Tan EH (2018). Afatinib as first-line treatment of older patients with *EGFR* mutation-positive non-small-cell lung cancer: subgroup analyses of the LUX-Lung 3, LUX-Lung 6, and LUX-Lung 7 trials. Clin Lung Cancer.

[43] Watanabe S, Yamaguchi OU, Masumoto AI (2018). Phase I study evaluating the combination of afatinib with carboplatin and pemetrexed after first-line EGFR-TKIs. Anticancer Res.

[44] Gazzah A, Boni V, Soria JC (2018). A phase 1b study of afatinib in combination with standard-dose cetuximab in patients with advanced solid tumours. Eur J Cancer.

[45] Ninomiya T, Nogami N, Kozuki T (2021). Survival of chemo-naive patients with *EGFR* mutation-positive advanced non-small cell lung cancer after treatment with afatinib and bevacizumab: updates from the Okayama Lung Cancer Study Group Trial 1404. Jpn J Clin Oncol.

[46] Shirley M (2018). Dacomitinib: First global approval. Drugs.

[47] Wu YL, Cheng Y, Zhou X (2017). Dacomitinib versus gefitinib as first-line treatment for patients with *EGFR*-mutation-positive non-small-cell lung cancer (ARCHER 1050): a randomised, open-label, phase 3 trial. Lancet Oncol.

[48] Nishio M, Kato T, Niho S (2020). Safety and efficacy of first-line dacomitinib in Japanese patients with advanced non-small cell lung cancer. Cancer Sci.

[49] Wu YL, Tsuboi M, He J (2020). Osimertinib in resected *EGFR*-mutated non-small-cell lung cancer. N Engl J Med.

[50] Mok TS, Wu Y, Ahn M (2017). Osimertinib or platinum-pemetrexed in EGFR T790M-positive lung cancer. N Engl J Med.

[51] Kishikawa T, Kasai T, Okada M (2020). Osimertinib, a third-generation EGFR tyrosine kinase inhibitor: A retrospective multicenter study of its real-world efficacy and safety in advanced/recurrent non-small cell lung carcinoma. Thorac Cancer.

[52] Sequist LV, Han JY, Ahn MJ (2020). Osimertinib plus savolitinib in patients with *EGFR* mutation-positive, *MET*-amplified, non-small-cell lung cancer after progression on EGFR tyrosine kinase inhibitors: interim results from a multicentre, open-label, phase 1b study. Lancet Oncol.

[53] Xu X (2015). Parallel phase 1 clinical trials in the US and in China: accelerating the test of avitinib in lung cancer as a novel inhibitor selectively targeting mutated *EGFR* and overcoming T790M-induced resistance. Chin J Cancer.

[54] Wang H, Zhang L, Hu P (2018). Penetration of the blood-brain barrier by avitinib and its control of intra/extra-cranial disease in non-small cell lung cancer harboring the T790M mutation. Lung Cancer.

[55] Ma Y, Zheng X, Zhao H (2018). First-in-human phase I study of AC0010, a mutant-selective EGFR inhibitor in non-small cell lung cancer: safety, efficacy, and potential mechanism of resistance. J Thorac Oncol.

[56] Yang JC, Camidge DR, Yang CT (2020). Safety, efficacy, and pharmacokinetics of almonertinib (HS-10296) in pretreated patients with *EGFR*-mutated advanced NSCLC: A multicenter, open-label, phase 1 trial. J Thorac Oncol.

[57] Shi Y, Zhang S, Hu X (2020). Safety, clinical activity, and pharmacokinetics of alflutinib (AST2818) in patients with advanced NSCLC with *EGFR* T790M mutation. J Thorac Oncol.

[58] Shi Y, Hu X, Zhang S (2021). Efficacy, safety, and genetic analysis of furmonertinib (AST2818) in patients with *EGFR* T790M mutated non-small-cell lung cancer: a phase 2b, multicentre, single-arm, open-label study. Lancet Respir Med.

[59] Jia Y, Yun CH, Park E (2016). Overcoming EGFR(T790M) and EGFR(C797S) resistance with mutant-selective allosteric inhibitors. Nature.

[60] Kashima K, Kawauchi H, Tanimura H (2020). CH7233163 overcomes osimertinib-resistant *EGFR*-Del19/T790M/C797S mutation. Mol Cancer Ther.

[61] Wu YL, Zhou C, Liam CK (2015). First-line erlotinib versus gemcitabine/cisplatin in patients with advanced *EGFR* mutation-positive non-small-cell lung cancer: analyses from the phase III, randomized, open-label, ENSURE study. Ann Oncol.

[62] Sequist LV, Martins RG, Spigel D (2008). First-line gefitinib in patients with advanced non-small-cell lung cancer harboring somatic *EGFR* mutations. J Clin Oncol.

[63] Mitsudomi T, Morita S, Yatabe Y (2010). Gefitinib versus cisplatin plus docetaxel in patients with non-small-cell lung cancer harbouring mutations of the epidermal growth factor receptor (WJTOG3405): an open label, randomised phase 3 trial. Lancet Oncol.

[64] Sequist LV, Waltman BA, Dias-Santagata D (2011). Genotypic and histological evolution of lung cancers acquiring resistance to EGFR inhibitors. Sci Transl Med.

[65] Campo M, Gerber D, Gainor JF (2016). Acquired resistance to first-line afatinib and the challenges of prearranged progression biopsies. J Thorac Oncol.

[66] Yun CH, Mengwasser KE, Toms AV (2008). The T790M mutation in EGFR kinase causes drug resistance by increasing the affinity for ATP. Proc Natl Acad Sci U S A.

[67] Zhao S, Li X, Zhao C (2019). Loss of T790M mutation is associated with early progression to osimertinib in Chinese patients with advanced NSCLC who are harboring EGFR T790M. Lung Cancer.

[68] Papadimitrakopoulou VA, Wu YL, Han JY (2018). LBA51 - Analysis of resistance mechanisms to osimertinib in patients with EGFR T790M advanced NSCLC from the AURA3 study. Ann Oncol.

[69] Patel H, Pawara R, Ansari A (2017). Recent updates on third generation EGFR inhibitors and emergence of fourth generation EGFR inhibitors to combat C797S resistance. Eur J Med Chem.

[70] Leonetti A, Sharma S, Minari R (2019). Resistance mechanisms to osimertinib in *EGFR*-mutated non-small cell lung cancer. Br J Cancer.

[71] Niederst MJ, Hu H, Mulvey HE (2015). The allelic context of the C797S mutation acquired upon treatment with third-generation EGFR inhibitors impacts sensitivity to subsequent treatment strategies. Clin Cancer Res.

[72] Arulananda S, Do H, Musafer A (2017). Combination osimertinib and gefitinib in C797S and T790M *EGFR*-mutated non-small cell lung cancer. J Thorac Oncol.

[73] Wang Z, Yang JJ, Huang J (2017). Lung adenocarcinoma harboring EGFR T790M and in trans C797S responds to combination therapy of first- and third-generation EGFR TKIs and shifts allelic configuration at resistance. J Thorac Oncol.

[74] Zhang M, Jang H, Nussinov R (2020). PI3K inhibitors: review and new strategies. Chem Sci.

[75] Eng J, Woo KM, Sima CS (2015). Impact of concurrent *PIK3CA* mutations on response to EGFR tyrosine kinase inhibition in *EGFR*-mutant lung cancers and on prognosis in oncogene-driven lung adenocarcinomas. J Thorac Oncol.

[76] Zhou X, Wang X, Zhu H (2021). PI3K inhibition sensitizes *EGFR* wild-type NSCLC cell lines to erlotinib chemotherapy. Exp Ther Med.

[77] Bean J, Riely GJ, Balak M (2008). Acquired resistance to epidermal growth factor receptor kinase inhibitors associated with a novel T854A mutation in a patient with *EGFR*-mutant lung adenocarcinoma. Clin Cancer Res.

[78] Costa DB, Schumer ST, Tenen DG (2008). Differential responses to erlotinib in epidermal growth factor receptor (*EGFR*)-mutated lung cancers with acquired resistance to gefitinib carrying the L747S or T790M secondary mutations. J Clin Oncol.

[79] Chiba M, Togashi Y, Bannno E (2017). Efficacy of irreversible EGFR-TKIs for the uncommon secondary resistant *EGFR* mutations L747S, D761Y, and T854A. BMC Cancer.

[80] Engelman JA, Zejnullahu K, Mitsudomi T (2007). *MET* amplification leads to gefitinib resistance in lung cancer by activating ERBB3 signaling. Science.

[81] Yu HA, Arcila ME, Rekhtman N (2013). Analysis of tumor specimens at the time of acquired resistance to EGFR-TKI therapy in 155 patients with *EGFR*-mutant lung cancers. Clin Cancer Res.

[82] Patil T, Mushtaq R, Marsh S (2020). Clinicopathologic characteristics, treatment outcomes, and acquired resistance patterns of atypical *EGFR* mutations and HER2 alterations in stage IV non-small-cell lung cancer. Clin Lung Cancer.

[83] Bean J, Brennan C, Shih JY (2007). *MET* amplification occurs with or without T790M mutations in *EGFR* mutant lung tumors with acquired resistance to gefitinib or erlotinib. Proc Natl Acad Sci U S A.

[84] Wu YL, Cheng Y, Zhou J (2020). Tepotinib plus gefitinib in patients with *EGFR*-mutant non-small-cell lung cancer with MET overexpression or *MET* amplification and acquired resistance to previous EGFR inhibitor (INSIGHT study): an open-label, phase 1b/2, multicentre, randomised trial. Lancet Respir Med.

[85] Jebbink M, de Langen AJ, Boelens MC (2020). The force of HER2 - A druggable target in NSCLC?. Cancer Treat Rev.

[86] Yarden Y, Sliwkowski MX (2001). Untangling the ErbB signalling network. Nat Rev Mol Cell Biol.

[87] Koga T, Kobayashi Y, Tomizawa K (2018). Activity of a novel HER2 inhibitor, poziotinib, for *HER2* exon 20 mutations in lung cancer and mechanism of acquired resistance: An *in vitro* study. Lung Cancer.

[88] Wang Y, Jiang T, Qin Z (2019). *HER2* exon 20 insertions in non-small-cell lung cancer are sensitive to the irreversible pan-HER receptor tyrosine kinase inhibitor pyrotinib. Ann Oncol.

[89] Yonesaka K, Takegawa N, Watanabe S (2019). An HER3-targeting antibody-drug conjugate incorporating a DNA topoisomerase I inhibitor U3-1402 conquers EGFR tyrosine kinase inhibitor-resistant NSCLC. Oncogene.

[90] Nakatani K, Yamaoka T, Ohba M (2019). *KRAS* and *EGFR* amplifications mediate resistance to rociletinib and osimertinib in acquired afatinib-resistant NSCLC harboring exon 19 deletion/T790M in EGFR. Mol Cancer Ther.

[91] Lin MW, Su KY, Su TJ (2018). Clinicopathological and genomic comparisons between different histologic components in combined small cell lung cancer and non-small cell lung cancer. Lung Cancer.

[92] Ho CC, Liao WY, Lin CA (2017). Acquired *BRAF* V600E mutation as resistant mechanism after treatment with osimertinib. J Thorac Oncol.

[93] Fassunke J, Muller F, Keul M (2018). Overcoming EGFR G724S-mediated osimertinib resistance through unique binding characteristics of second-generation EGFR inhibitors. Nat Commun.

[94] Offin M, Somwar R, Rekhtman N (2018). Acquired *ALK* and *RET* gene fusions as mechanisms of resistance to osimertinib in *EGFR*-mutant lung cancers. JCO Precis Oncol.

[95] Schrock AB, Zhu VW, Hsieh WS (2018). Receptor tyrosine kinase fusions and BRAF kinase fusions are rare but actionable resistance mechanisms to EGFR tyrosine kinase inhibitors. J Thorac Oncol.

[96] Yu HA, Arcila ME, Rekhtman N (2013). Analysis of tumor specimens at the time of acquired resistance to EGFR-TKI therapy in 155 patients with *EGFR*-mutant lung cancers. Clin Cancer Res.

[97] Lee J K, Lee J, Kim S (2017). Clonal history and genetic predictors of transformation into small-cell carcinomas from lung adenocarcinomas. J Clin Oncol.

[98] Dongre A, Weinberg RA (2019). New insights into the mechanisms of epithelial-mesenchymal transition and implications for cancer. Nat Rev Mol Cell Biol.

[99] Du W, Sun L, Liu T (2020). The miR-625-3p/AXL axis induces non-T790M acquired resistance to EGFR-TKI via activation of the TGF-β/Smad pathway and EMT in *EGFR*-mutant non-small cell lung cancer. Oncol Rep.

[100] Zhu X, Chen L, Liu L (2019). EMT-mediated acquired EGFR-TKI resistance in NSCLC: mechanisms and strategies. Front Oncol.

[101] Yang JC, Gadgeel SM, Sequist LV (2019). Pembrolizumab in combination with erlotinib or gefitinib as first-line therapy for advanced NSCLC with sensitizing *EGFR* mutation. J Thorac Oncol.

